# Rapid Fabrication of Disposable Micromixing Arrays Using Xurography and Laser Ablation

**DOI:** 10.3390/mi8050144

**Published:** 2017-05-04

**Authors:** J. Israel Martínez-López, H.A. Betancourt, Erika García-López, Ciro A. Rodriguez, Hector R. Siller

**Affiliations:** Tecnológico de Monterrey, Eugenio Garza Sada 2501 Sur, 64849 Monterrey, NL, Mexico; habc-8@hotmail.com (H.A.B.); garcia.erika@itesm.mx (E.G.-L.); ciro.rodriguez@itesm.mx (C.A.R.); hector.siller@itesm.mx (H.R.S.)

**Keywords:** micromixing, split and recombine, rapid manufacture, xurography, laser ablation

## Abstract

We assessed xurography and laser ablation for the manufacture of passive micromixers arrays to explore the scalability of unconventional manufacture technologies that could be implemented under the restrictions of the Point of Care for developing countries. In this work, we present a novel split-and-recombine (SAR) array design adapted for interfacing standardized dispensing (handheld micropipette) and sampling (microplate reader) equipment. The design was patterned and sealed from A4 sized vinyl sheets (polyvinyl chloride), employing low-cost disposable materials. Manufacture was evaluated measuring the dimensional error with stereoscopic and confocal microscopy. The micromixing efficiency was estimated using a machine vision system for passive driven infusion provided by micropippetting samples of dye and water. It was possible to employ rapid fabrication based on xurography to develop a four channel asymmetric split-and-recombine (ASAR) micromixer with mixing efficiencies ranging from 43% to 65%.

## 1. Introduction

The development and spread of advanced diagnostic devices based on microfluidic technology can be a key strategy element to tackle a wide variety of infectious diseases such as AIDS, tuberculosis (TB) or malaria. Despite the availability of diagnostics and solutions for these diseases, every year about 15 million people die from these diseases [1]. Academics have proposed a wide variety of devices built on advanced manufacturing, electromechanical sensors and actuators, and Information and Communications Technologies (ICT) to treat patients outside the boundaries of a hospital. A significant portion of these devices, gadgets, and systems had been conceived to serve in the Point of Care (POC). The approach to bringing healthcare closer to the patients is inherently well encompassed with technologies that enable the miniaturization of established diagnostics, treatment, and monitoring illness. In developing countries, mobility is an asset that can enhance aid by providing closer access to remote areas, treating illnesses at earlier stages, and deterring the spread of infectious diseases.

The World Health Organization (WHO) has resumed the requirements of the in-field solutions under the acronym “ASSURED”, which stands for affordable, sensitive, specific, user-friendly, rapid and robust, equipment-free or minimal equipment, and deliverable to end-users. The design and manufacture of POC devices carries added demanding development requirements. This equipment should remain functional in its main purpose reliably under the constraints of being low-cost, the absence of trained staff, lack of electricity, poorly equipped laboratory facilities, and limited access to refrigeration and storage [2,3]. Studies evaluating the performance of POC prognosis have shown that end-users (typically remote health workers or volunteers) are affected by some factors, including manual dexterity, visual acuity, and available lighting during testing. Micropipette or other standardized methods for sample management and transfer can support a more reliable and high-throughput capable device operation [4,5].

Despite the copious amount of research in the design and operation of micromixers, research regarding manufacture technology towards implementation beyond academic environments is limited. Microfabrication-based on the photolithographic processes of polymers is the most common approach employed [6]. Typically, this implies procedures that require supervision by specialized personnel under laboratory facilities (i.e., spin coaters, ovens, plasma treatment, hot plates) and requires the supply of resins for the development of structures within the finesse in the micrometric scale. These specimen can then be used as sample devices or employed to replicate them through casting, stamping, or injection molding. Polydimethylsiloxane (PDMS) is the most popular elastomer due the ability to cast with nanometric resolution and to be relatively inexpensive and because it can be irreversibly bonded to other materials such as glass or other polymeric films [7,8].

In rapid fabrication manufacturing techniques, devices are fabricated faster than conventional manufacturing processes. While some of these methodologies were originally conceived solely for producing one or few samples within some surface or structural quality limitations, now it is possible to find cases where these techniques have evolved into the production of components that could not be made otherwise [9]. The incorporation of rapid fabrication technology for microfluidic devices is a growing trend among researchers, but it has not yet been fully developed [10]. 3D printing has raised much awareness among academics and media because it allows devices to be manufactured on-demand with ease and quickness for medical applications [11] of microfluidic devices [12].

Compared to other rapid fabrication technologies such as fused deposition modeling (FDM) or stereolithography (SL), technology that relies on processing thin-films rolls is typically more affordable and can be transported more easily than liquid-based reagents like PDMS or the resins used for the processes mentioned above. The roll to roll hot embossing process is a recent advancement in micro hot embossing processes and is capable of continuously fabricating micro/nano structures on the polymer with high efficiency and high throughput [13,14]. High volume fabrication employing manufacture based on processing polymer on rolls has been used successfully for pinched flow fractionation on cellulose acetate [13]. Another group developed an electrophoresis chip for the detection of antibiotic resistance bacteria by feeding a thermoplastic foil through a hot embossing cylinder [15]. Senkbeil et al. have altered the rheological behavior of the resin system to produce capillary electrophoresis chips.

Xurography is a rapid manufacture technology that originated from the adaptation of equipment intended for advertisement used for the development of microfluidic systems. It relies on the patterning and removal of thin-films materials using a blade tracing a design [16,17]. Originally, around a decade ago, the major advantage was the simplicity and quickness in operation; nowadays, the cost reduction in these equipment from the original 4000 USD to 200 USD has accentuated the price-value ratio advantage of this technique compared to more conventional approaches. For example, Silhouette [18] and Cricut [19] are two providers that offer desktop sized cutting equipment designed for home use with a starting price of 200 USD for processing standardized A4 sheets with comparable cutting performance to more expensive equipment.

Another technique used for manufacturing microdevices from thin layers of materials formatted as rolls is laser ablation. Some research has been developed widely in PDMS [20], glass [21,22], cyclic olefin copolymer (COP) [23], and polymethyl methacrylate (PMMA) [24,25] materials with a variety of applications in lab on a chip field. These microdevices have been processed using an ultra short pulsed laser (e.g., femtosecond and picosecond pulse durations), which results in a promising technique in micromachining that relies on design flexibility, precision, and productivity. Laser ablation for microdevices is performed through the interaction between laser energy and the material, where the main parameters are wavelength and pulse duration. Therefore a focused volume absorbs laser energy, which allows a localized machining, while the rest of the sample results are unaffected [26]. Among the laser ablation equipment, engravers are a subset of machines suited for cutting non-metallic materials. Prices can vary widely depending on the type of laser, work area, and features from roughly 8000 USD to 5000 USD. Recently there have been some efforts funded by crowdfunded projects to commercialize prototypes focused on hobbyists with lower price baselines and added features [27,28]. However these alternatives remain above 500 USD and are still in development.

One of the desirable operations that a POC device must perform is micromixing since devices at the micrometric scale tend naturally to operate on a laminar flow regime. Under that condition, the homogenization and reaction of reagents tend to be slow or require systems with large characteristic length to function properly [29,30]. Rather than perform micromixing by a supplementary force, passive micromixing depends on geometrical features along the flow chamber. For example, slanted grooved [31,32] and staggered herringbone [33] micromixers induce homogenization by creating secondary flows using obstacles or other complex features along the flow chamber. These designs are typically highly efficient in the task of mixing but also require expensive multi-layer manufacture technology.

A widely studied example of a passive micromixer is the T-mixer. In this simple design, two separate fluids are brought into contact from opposite directions and then leave through a channel that is perpendicular to the inlet channels [24]. The performance tends to be low as the mixing occurs only proximate to the junction. A more recent methodology, SAR or ASAR micromixers force this contact by repeatedly putting together the streams from the inputs and hence increasing the interfacial area of the streams. Recent research had employed the principle of the T-mixer by splitting and recombining streams iteratively with more complex geometry as rhombic [34,35], modified Tesla [36,37,38], or curve based [39,40,41] and shapes based setups. Table 1 represents the recent development and the manufacture methodologies and some micromixers channels per device (*N*) for in-plane micromixers prior to this work. It is noticeable that, before the present work, Chung et al. [42] used a laser as part of a manufacturing process, but this was not done with consideration of either POC or rapid manufacturing. The authors have not found previous work from other research groups on the development of interfacing a micromixer with standardized sample management equipment, neither have they found deported efforts to develop full-sized arrays.

To address these restrictions in the deployment of a particular type of microfluidic device (SAR) micromixer, we have recently developed a methodology to produce single devices from scratch to testing without the requirement of ancillary laboratory equipment [10,45]. Combining xurography and lamination offered promissing advantages over conventional manufacture such as flexibility, short cycle times, and low-cost. However, to deploy micromixers such as POC, it is important to confront the scalability of the process to produce massively and reliably these devices in the field.

In this work, we present a scaled-up version of a novel split-and-recombine (SAR) array design presented before by our group [10,45] and adapted to be an interface for a handheld multichannel micropipette as a step forward to meet the aforementioned criteria. The manufacturability has been assessed for xurography and laser ablation. A benchmark between these manufacturing processes is relevant as they are the commercially available alternatives for processing thin-films without specialized laboratory equipment.

While the mere availability of POC technologies does not automatically ensure their adoption [46,47], the authors of this article believe that the flexibility of rapid fabrication provided by manufacture based on polymeric films along high-throughput capabilities can ease the integration of micromixing as part of more complex and functional diagnosis, treatment, and monitoring systems. For example, micromixing has shown potential as a low-cost sensitivity enhancer for biosensing micro-devices [48,49,50].

## 2. Materials and Methods

A Graphtec CE5000–60 (Graphtec America, Irvine, CA, USA) high precision cutting plotter and a Telesis EV25DS (Telesis Technologies, Circleville, OH, USA) marking system were used to assess xurography and laser ablation. Standard commercial Arlon vinyl sheets (polyvinyl chloride; Arlon Graphics, Placentia, CA, USA) were used for the manufacturability assessment (this vendor was selected considering the availability of a worldwide distributor network). Manufacture using xurography was employed on a testing material with three color variations; gray (G_X_), orange (O_X_), and black (B_X_) using a similar methodology introduced by a previous work for a single micromixer device [10,45] and then adapted and employed on materials of the same batch for gray (G_L_), orange (O_L_), and black (B_L_) using ablation. The procedures can be summarized as follows: a microfluidic device can be developed by patterning and stacking four layers of materials. A PMMA substrate (Layer 0) provides the mechanical stiffness required for handling the device, a vinyl pattern (Layer 1) forms the flow cell walls, and an acetate sheet (Layer 2) is employed to provide an enclosure ceiling to the device and provide additional structural support to the (Layer 4) vinyl sheet that seals the device and delimits the inlets and outlets. Table 2 and Table 3 and Section 2.1 and Section 2.2 describe more thoroughly the setup conditions.

**Figure 1:**
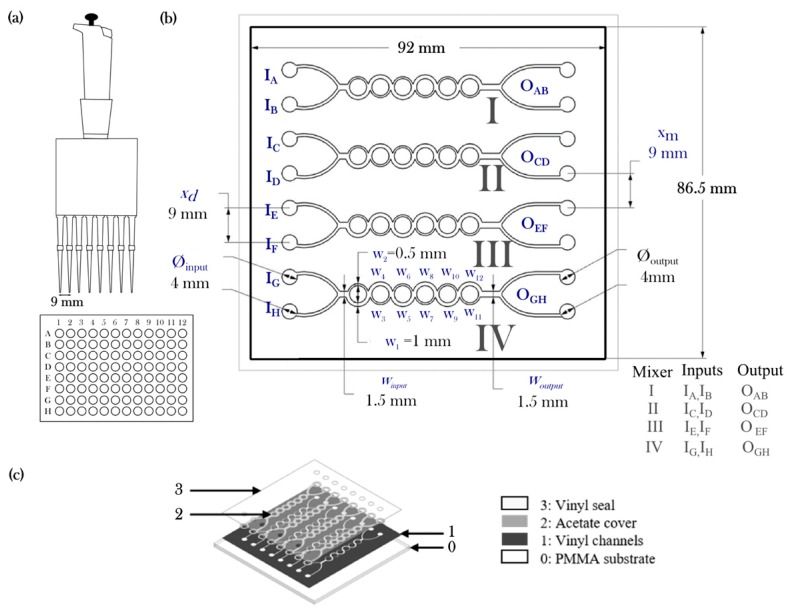
(**a**) Schematics of standardized handheld multichannel micropipette and microwell plate, (**b**) four-channel asymmetric split-and-recombine (ASAR) micromixer (two inputs and two outputs) microdevice setup used to evaluate xurography and laser ablation, (**c**) conforming layers of a microdevice.

The implementation of the rapid fabrication methodology studied in this article was assessed in the manufacturing process of a passive micromixing following a procedure presented in previous work [10] and expanded for an array configuration. The implemented design is based on previous research [10,40,45] on an unbalanced split and recombine micromixer. Figure 1a,b shows our proposed testing device setup. A standard 96 microwell microplate (Sigma-Aldrich, Saint Louis, MO, USA) or microtiter is used to store samples prior processing. A standard multichannel micropipette (Thermo Fischer, Waltham, MA, USA) was used to collect the samples from the microwells to introduce them in a four channel microdevice (I to IV). Figure 1b describes the array configuration: each of the channels is integrated by two inputs (*I*_A_, *I*_B_, *I*_C_, etc.), a six phase asymmetric split, and a recombine micromixer; whereas the input main channel (*w*_input_ = 1500 μm) is divided subsequently into a main subchannel (*w*_1_ = *w*_3_ = *w*_5_ = *w*_7_ = 1000 μm) and a lesser subchannel (*w*_2_ = *w*_4_ = *w*_6_ = *w*_8_ = *w*_10_ = *w*_12_ = 500 μm). The inputs and outputs of the device (⌀_input_ = ⌀_output_) are equally spaced (*x*_d_ *= x*_m_ = 9 mm) and share the same dimension pipette to pipette distance. The overall size of the micromixer array is 92 mm × 86.5 mm.

### 2.1. Xurography Setup

A Graphtec CE5000-60 (Graphtec America, Irvine, CA, USA) high precision cutting plotter was used to pattern 4500 CalPlus vinyl sheet (Layer 1, Arlon Graphics LLC, Placentia, CA, USA). This equipment has a list price around $2000 USD.

Figure 1b describes the four layers composing the micromixing arrays. Commercial acetate sheets transparency foils were used to pattern the intermediate layer (Layer 2; 21.59 × 27.94 cm). The thickness of the acetate sheets was found to be variable among the stock and was examined using confocal microscopy prior experimentation. Devices were sealed using a CalPlus 5000 transparent polymeric film (Layer 3, Arlon Graphics LLC, Placentia, CA, USA) to the cutting machine software (Graphtec Design Studio, Graphtec America, Irvine, CA, USA) for patterning Layers 1, 2, and 3. The cutting tool used for this work was a standard carbide cutting tool model CB09U with a cutting diameter of 0.9 mm and a cutting angle of 45°. Table 2 and Table 3 describe the details of the setup conditions for patterning the materials.

### 2.2. Laser Ablation Setup

Untreated Arlon vinyl sheets (100 mm × 100 mm) with the same features mentioned in Table 2 were adhered to commercial PMMA sheets (Layer 0). A fiber-coupled diode end pumped Q-switched Nd: YVO_4_ laser was used for patterning the micromixing array. Table 2 presents the main features of the Telesis equipment employed in the experimental trials. This machine is an industrial laser engraver with a list price around $46,000 USD, including hardware and software. The laser beam was focused on vinyl sheets using a 420 mm focal lens and a galvo-mechanism resulting in a theoretical spot size of 127 µm. Trajectories were defined according to a DXF file performed in Autocad (Autodesk, Mill Valley, CA, USA), and marking parameters were established on Merlin II LS software (Telesis, Circleville, OH, USA). The assembly process was made as follows; once the laser ablated the vinyl material (Layer 1) from the PMMA substrate (Layer 0), a tweezer was used to separate the channel geometry from the PMMA substrate (Layer 0). Layer 2 and Layer 3 were added subsequently manually in a similar manner as the samples processed with xurography.

### 2.3. Array Characterization

Distilled water and food colorant (blue) were used for the visual inspection of the microdevices for the detection of leaks. Microdevice characterization was carried out with an SV8 Zeiss stereoscopic microscope (Carl Zeiss Microscopy, Cambridge, UK) to measure the microdevice dimensions (channel widths and lengths). To evaluate the quality of the patterning process among the conditions, micromixing array elements and features were measured with two replicas. In total, 192 measurements were made to assess the patterning process quantitatively. To compare the dimensional errors between xurography and laser ablation, the absolute average dimensional error was employed, which comprises the percentage error of the undercuttings and overcuttings by considering the mean of the absolute values of the differences between the experimental and intended feature sizes expressed in a percentage.

A confocal microscope Axio CSM 700 (Carl Zeiss Microscopy) was employed to determine the depth of the microdevices during the different steps of the lamination process.

### 2.4. Micromixing Characterization

The examination and quantification of the micromixing performance were assessed using a custom made machine vision system. Dilutions of a food color additive and distilled water samples were prepared and placed in an intercalated order in a microwell and then transferred into the device by releasing 40 μL droplets of the samples over the inputs of the channel using a multichannel micropipette. Distilled water and a standard compressed air duster (Office Depot, Boca Raton, FL, USA) were used to wash the devices before and after the assessment. The food color additive was composed of water, glycerin, and corn syrup.

To quantify the mixing behavior, the variance of the liquid species in the micromixer (*σ*) was calculated based on the Danckwerts’ segregation intensity index [51]. The variance of the mass fraction of the mixture in a cross-section (*σ*) that is normal to the flow direction is defined as follows:

(1) $$\sigma =\sqrt{\frac{1}{N}{\left(I_{\mathrm{ni}}-\bar{I_{\mathrm{nm}}}\right)}^{2}},$$

where *N* is the number of sampling points inside the cross section, $I_{\mathrm{ni}}$ is the normalized intensity value at pixel *I*, and $\bar{I_{\mathrm{nm}}}$ is the normalized mean intensity value at the target area. To quantitatively analyze the numerical mixing performance of the micromixer, the mixing index (M) at a cross-sectional plane is defined as:

(2) $$M=1-\sqrt{\frac{\sigma^{2}}{{\sigma_{\max}}^{2}}}$$

where the mixing efficiency ranges from 0.00 (0% mixing) to 1.00 (100% mixing). The maximum variance *σ*_max_ represents a completely unmixed condition ($\sigma_{\max}=0.5)$. The target areas (138 × 40 pixels) were defined at each output of the channels of the micromixers to extract intensity mean and variance values. Considering the non-uniform flow derived from the tensional passive drive flow produced by the multi-channel micropipette, the intensity was normalized with the concentration of the blue dye.

A C930 Logitech digital camera (Logitech International, Lausanne, Switzerland) with 1920 × 1080 pixels and a 90-degree field of view was employed to capture the images for processing. Mixing quantification was done using a custom made software using Visual Basic (Microsoft Corporation, Redmond, WA, USA) and Aforge.NET, an open source framework for researchers in Computer Vision and Artificial Intelligence [52].

## 3. Results and Discussion

**Figure 2:**
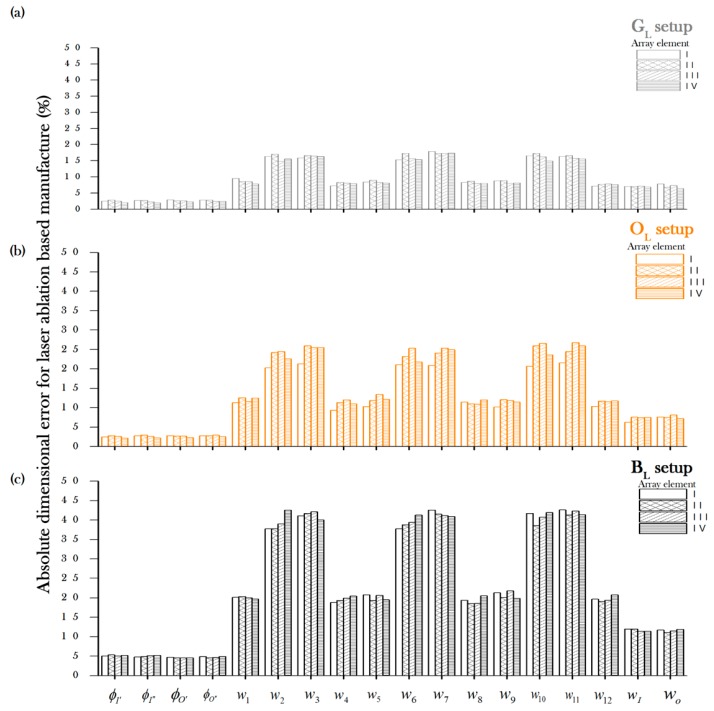
Absolute deviation of the geometrical features of an asymmetric split-and-recombine (ASAR) micromixer for a laser ablation-based manufacture process for (**a**) gray vinyl (G_L_), (**b**) orange vinyl (O_L_) and (**c**) black vinyl (B_L_).

**Figure 3:**
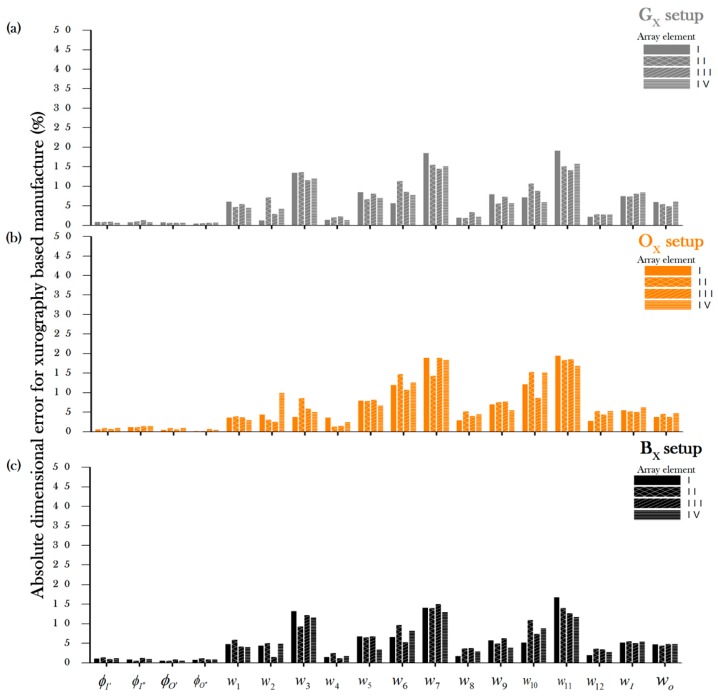
Absolute deviation of the geometrical features of an asymmetric split-and-recombine (ASAR) micromixer for a xurography-based manufacture process for (**a**) gray vinyl (G_X_), (**b**) orange vinyl (O_X_) and (**c**) black vinyl (B_X_).

Assessment of the rapid manufacturing techniques showed that both manufacturing techniques were tested for different materials and setups (see Table 2 for details) to evaluate the absolute deviational error along the micromixer array elements (from I to IV) of each of the features. Figure 2 and Figure 3 describe the average absolute errors for laser ablation and xurography, respectively. The error is comprised by undercutting and overcutting for each of the setup conditions during the patterning of the gray (G_L_,G_X_), orange (O_L_,O_X_), and black (B_L_,B_X_) materials. The measure of the depth of the conforming Layer 1 and hence the depth of the micromixer (*d*_m_) were consistently found to be approximately 100 μm, regardless of the color of the material. The magnitude of the deviational errors varied greatly depending on the type of feature. For example, inputs and outputs (⌀_input_ and ⌀_output_) were processed consistently with errors below 5%, the wider main channels (*w*_input_ and *w*_output_*)* and the main subchannels showed higher errors (from 5% to 12%), and the most finessed regions of the device such as the minor subchannels (*w*_1_, *w*_3_, *w*_5_, *w*_7_, *w*_11_) displayed the highest deviational errors with values up to 42%. It should be mentioned that, for all the patterning setups, the differences among channels was low, suggesting that the precision of the channel is suitable for further scaling up of the number of micromixing channels. Among all the setups, the G_L_ configuration (gray vinyl under laser ablation) showed the lowest device to device variability (standard deviation) among the measurements.

Technological considerations arose during the assessment of the laser ablation experimentation as follows. The results indicate an absolute dimensional error that was larger in lesser channels, which is explained by heat affected zones where thermal diffusivity is promoted in narrow zones (e.g., 500 µm subchannels) and consequently cut widths with greater variability. Also, the differences among orange, black, and gray setups are explained due to the coefficient absorption of Nd: YVO_4_ by differences among the optical properties of the materials. The aforementioned condition explains the steep differences in quality performance among the laser ablation setups (Figure 2) and the similar overall performance among xurography setups. Moreover, the use of laser engraving or laser cutting systems in industrial settings can lead to a regular exposure of particles generated during material processing. For the assessment, we had considered a report on the risks of exposure of polyvinyl chloride in an industrial setting [53] to carry out an evaluation in an enclosed chamber. While it is still necessary to determine the magnitude of the risks for human health and security, the circumstance adds a limitation layer for the utilization of laser ablation, especially aiming towards high-throughput performance for the POC setting.

The absolute dimensional error for then xurography based manufacturing process (see Figure 3) was shown to be below 5% for the circular patterns for the inputs and the outputs and higher among the features with finer and more complex details such as the the minor subchannels (*w*_2_, *w*_3_, *w*_6_, *w*_7_, *w*_10_, *w*_11_). Differences in performance among the array elements can be attributed to uneven conditions of the material properties of the surface, errors in positioning derived from the moving mechanical parts, and the gradual loss of sharpness of the blade.

**Figure 4:**
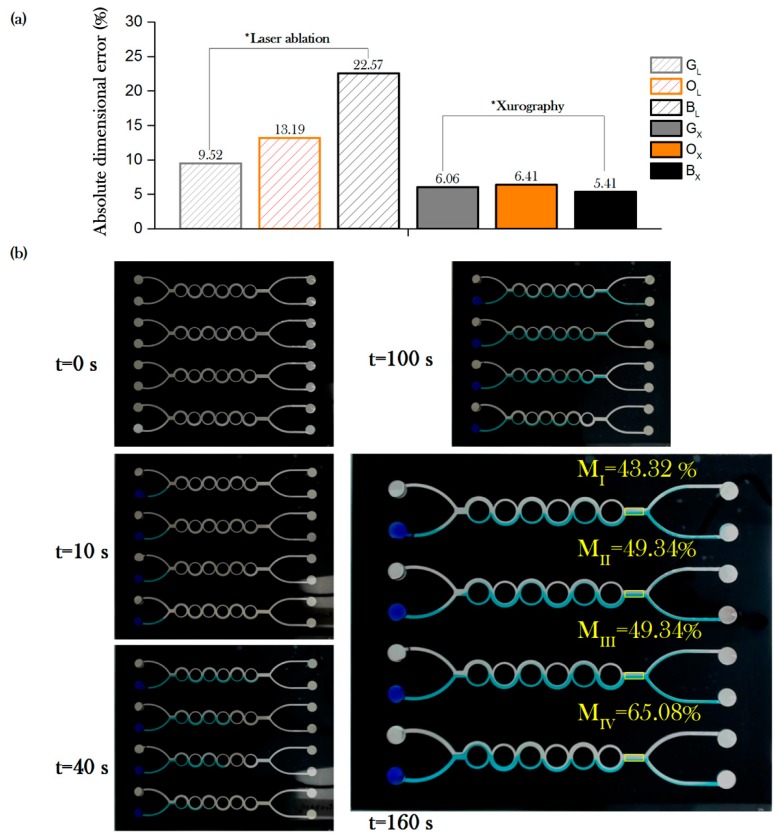
(**a**) Overall absolute deviational error for laser ablation and xurography based manufacture; (**b**) example of a four-channel device mixing after *t* = 0, 10, 40, 100, and 160 s of passive driven flow, mixing efficiencies are indicated in yellow for each of the array elements.

The overall performance of laser ablation and xurography is shown in Figure 4a. Setup B_X_ (black vinyl under xurography) showed among all the setups the highest quality, with only 5.41% absolute deviational error. For the laser ablation, we were able to manufacture the array of devices within comparable values from previous research for the G_L_ setup; however the quality of the orange O_X_ and B_X_ setups suggest that improving the quality of Layer 1 of the device requires optimization using some of the other laser ablation parameters.

To confirm the B_X_ setup as a suitable manufacture methodology for a multi-channel ASAR micromixer array, an evaluation of the mixing performance was done following the procedure described in Section 2.3. Figure 4b shows pictures of 0, 10, 40, 100, and 160 s after the sample was introduced on the inlets simultaneously using the multichannel micropipette. The micromixing efficiency was estimated at the output of the channels. A video of the experimental test can be found in a data archiving service and as Supplementary Material (video S1: Micromixing test of a four channel split and recombine array) [54].

The Reynolds number is conventionally used to characterize the fluidic behavior in microdevices and is defined as the ratio of inertial to viscous forces. Equation (3) represents the Reynolds number (*Re*), defined as:

(3) $$Re=\frac{\mathrm{inertial}\;\mathrm{force}}{\mathrm{viscous}\;\mathrm{force}}=\frac{\rho UD_{H}}{\mu }.$$

where *µ* is the viscosity(Pa∙s), ρ is the fluid density (kg∙m^−3^), *U* is the average velocity of the flow (m∙s^−1^), and *D*_H_ is the hydraulic diameter of channel (m), which is defined in Equation (4) as:

(4) $$D_{H}=\frac{4A}{P}=\frac{4wd_{m}}{2w+2d_{m}},$$

where *A* and *P* are, respectively, the area and the wetted perimeter of the cross-section, which is given by the micromixer width (*w* *= w*_input_) and depth of the channel (*d*_m_).

The water properties at 20 °C density (*ρ*) were considered in 9.998 × 10^2^ kg∙m^−3^ and the dynamic viscosity (*σ*) in 1.01 × 10^−3^ kg∙m^−1^∙s^−1^ [32]. The mixing efficiency was evaluated using the Danckwerts’ segregation intensity index (see Section 2.2) 160 s after the samples were introduced in the microdevice. To estimate the Reynolds number and hence determine the flow regime in which each of the micromixers array elements is operating under the flow velocity, *U* was estimated by measuring the time required for the dye to reach the output (interrogation window).

Mixing evaluation windows are indicated in yellow at the outputs of the mixing array micromixers elements at *t* = 160 s. The Reynolds number was estimated ranging from 0.07 to 0.13, with corresponding mixing efficiency varying from 43.32% up to 65.08% (see Table 4). While the lateral dimensions of the microfluidic devices are larger than other in-plane micromixers reported in the literature (see Table 1), the flow regime remains laminar considering the device remains operating below 2300. To determine the source of the differences among the flow velocities between the array elements requires further experimental work. Differences in the average flow velocity are prone to arise due to various factors including the geometrical features of the channel produced by errors during the patterning and assembly of the conforming layers, non-uniform dispense of the liquid samples during pipetting, and clogging caused by debris or bubbles located inside the flow chamber during experimentation.

## 4. Conclusions and Future Work

- The dimensional accuracy of xurography was shown to be better for xurography than laser ablation for the ASAR micromixing array. Compared to xurography, the deployment of the laser ablation as a manufacturing tool in the POC setting underwent several disadvantages such as the requirement to adjust the setup parameters regarding the optical properties of the material and the additional health and security considerations for the laser processing of materials.
- Assessments of both the rapid manufacture technologies were successfully employed to produce low-cost microfluidic device arrays with deviational errors below 10% under certain setup conditions for xurography and laser ablation.
- Small differences in the dimensional errors among different ASAR micromixer members suggests that it is possible to scale-up further the size of the array.
- The proposed four element micromixer array design was successfully coupled with a standardized multichannel micropipette for micromixing simultaneously eight samples of dye with mixing performance up to 65%.
- The proposed design interfaces standardized dispensing (handheld micropipette) and sampling (microplate well) equipment.
- In the future, it is necessary to validate the mixing performance of the micromixing devices under different conditions (materials, geometries, instrumentation setup). Additional research is also required to determine factors affecting the systematic dimensional errors found in certain components of the micromixing device.

## Figures and Tables

**Table 1:** Manufacture methodologies for in-plane micromixers.

Work	Reference	Manufacture Methodology	*N*	*w*_input_
Hong et al. (2004)	[37]	Molding (nickel-SU-8), photolitography, hot embossing, drilling, thermal bonding	1	200 μm
Sudarsan & Ugaz (2006)	[39]	Circuit printing, etching, heat treatment	1	150 μm
Chung & Shi (2007)	[34]	Lithography, micro-molding, oxygen plasma treatment bonding, mechanical punching	1	500 μm
Chung et al. (2009)	[42]	Laser machining, PDMS casting from PMMA, thermal and oxygen plasma bonding, mechanical punching	1	500 μm
Ansari et al. (2010)	[40]	SU-8 photolithography over a silicon wafer, PDMS molding, mechanical punching	1	300 μm
Scherr et al. (2012)	[43]	SU-8 photolithography, PDMS molding, plasma cleaning, mechanical punching	1	30–200 μm
Li et al. (2013)	[44]	PDMS molding	1	300 μm
Martínez-López et al. (2016)	[10]	Xurography of PVC and manual lamination	1	750 μm

**Table 2:** Setup conditions for laser ablation and xurography.

Setup	Manufacture Technology	Patterning Mechanism	Patterning Conditions	Testing Material
G_X_,O_X_,B_X_	Xurography: Graphtec CE5000-60	Blade CB09U (45°)	Fload ≈ 0.8 N, Number of passes = 1	Gray, Orange, Black 4500 CalPlus
G_L_,O_L_,F_L_	Laser ablation: Telesis EV25DS	Q-switched Nd: YVO_4_ laser	Mark speed = 500 mm/min, Frequency = 10 kHz, Laser power = 22.5 W, Pass number = 10	Gray, Orange, Black 4500 CalPlus

**Table 3:** Machine setup conditions for the laser ablation process.

Condition	Specification
Laser type	Class 4, fiber-coupled, diode-pumped, Q-switched Nd: YVO_4_
Wavelength	1064 nm
Mode	TEM_00
Cooling system	Air-cooled
Galvanometer repeatibility	<22 micro radian
Field resolution	16 bit (65,535 data points)
Marking field size (420 mm lens)	290 × 290 mm

**Table 4:** Flow conditions and mixing efficiency of a four channel micromixer device manufactured using xurography.

Mixing Array Element	Average Flow Velocity (*U*)	Reynolds Number (*Re*)	Mixing Efficiency (*M*)
I	0.7 mm/s	0.13	43.32%
II	0.5 mm/s	0.09	49.34%
III	0.47 mm/s	0.08	49.34%
IV	0.38 mm/s	0.07	65.08%

## Supplementary Materials

The following is available online at www.mdpi.com/2072-666X/8/5/144/s1, Video S1: Micromixing test of a four channel split and recombine array.

## Abbreviations

The following abbreviations are used in this manuscript:

AIDS	Acquired Immune Deficiency Syndrome
ASAR	Asymmetric split and recombination
CAD	Computer aided design
COC	Cyclic olefin copolymer
COP	Cyclic olefin polymer
DXF	Drawing Interchange Format
EP	Electrophoresis
FDM	Fused deposition modeling
SAR	Split and recombine
SGM	Slanted grooved mixer
SHM	Staggered herringbone mixer
PC	Polycarbonate
PET	Polyethylene terephthalate
PETG	Polyethylene terephthalate glycol
PDMS	Polydimethylsiloxane
PEEK	Polyether ether ketone
PMMA	Polymethyl methacrylate
POC	Point-of-Care
PVC	Polyvinyl chloride
RGB	Red-green-blue
SL	Stereolitography
TB	Tuberculosis
WHO	World Health Organization
